# Preclinical activity of EGFR and MEK1/2 inhibitors in the treatment of biliary tract carcinoma

**DOI:** 10.18632/oncotarget.10587

**Published:** 2016-07-13

**Authors:** Giuliana Cavalloni, Caterina Peraldo-Neia, Chiara Varamo, Giovanna Chiorino, Francesco Sassi, Massimo Aglietta, Francesco Leone

**Affiliations:** ^1^ Medical Oncology Division, Fondazione del Piemonte per l'Oncologia (FPO), Candiolo Cancer Institute IRCCS, Candiolo, Italy; ^2^ Department of Oncology, University of Turin, Candiolo Cancer Institute IRCCS, Candiolo, Italy; ^3^ Cancer Genomics Laboratory, Fondazione Edo ed Elvo Tempia Valenta, Biella, Italy; ^4^ Unit of Molecular Pharmacology, University of Turin Medical School, Candiolo Cancer Institute IRCCS, Candiolo, Italy

**Keywords:** biliary tract carcinoma, K-RAS mutation, target therapy, MEK inhibitor, preclinical models

## Abstract

Biliary tract carcinomas (BTC) are malignant tumors with limited therapeutic options. Clinical experiences with anti-EGFR therapies have produced unsatisfactory results. The strategies of combined inhibition of EGFR and MEK1/2 could be a promising therapeutic option in BTC treatment. Preclinical activity of Panitumumab and Trametinib was tested in *in vitro* (EGI-1, MT-CHC01 and WITT cells) and in *in vivo* (xenograft) BTC models with different K-RAS mutational status. Trametinib reduced MAPK phosphorylation in wild type (WT) WITT cells and in both K-RAS mutated cells; in EGI-1 was also able to switch off EGFR activation. Panitumumab reduced the activation of its target only in EGI-1 cells, and of MAPK only in WITT cells. While Trametinib inhibited cell growth in K-RAS mutated cell lines, Panitumumab had no effect on proliferation independently by K-RAS status. The addition of Panitumumab to Trametinib did not significantly potentiate its anti-proliferative effect also in mutated cells. *In vivo*, Trametinib was able to significantly slow the tumor growth in K-RAS mutated xenograft models, but did not have effect on K-RAS WT cells; the addition of Panitumumab potentiated the Trametinib efficacy in MT-CHC01 and overcame the resistance to the anti-EGFR in WITT cells, in which the monotherapy was ineffective. Only in K-RAS mutated xenografts Trametinib alone or in combination with Panitumumab significantly decreased Ki67 positive cell fraction and CD31 angiogenesis markers. In conclusion, this preclinical study provides a rational to plan clinical trials assessing the efficacy of Trametinib in K-RAS mutated BTC patients and the combination with anti-EGFR in WT BTC patients.

## INTRODUCTION

Biliary tract carcinoma (BTC) is a highly and heterogeneous malignant epithelial neoplasm arising either from the intrahepatic or from the extrahepatic biliary tract and gallbladder [1]. Surgical resection is the only curative approach, although the majority of patients has an advanced unresectable disease at diagnosis; further, most patients surgically treated develops tumor recurrence [2, 3]. Therefore, it is urgent to find new and more effective therapies, possibly based on a better understanding of the molecular pathogenesis of these tumors.

The expression of EGFR is common in BTC, but an overexpression was described in a wide range (5–32%) of cases and is more frequently in intrahepatic cholangiocarcinoma (ICC) [4–6]. Activating mutations of K-RAS and B-RAF can occur in biliary cancers, with and incidence ranging from of 8% to 58% and 0 to 22% respectively, suggesting that the activation of the RAF/MAPK signaling pathway may be one of the key event in BTC carcinogenesis [7–12].

Recently, several reports demonstrated that anti-EGFR based therapies combined with chemotherapy marginally improve the outcome of these tumors. The combination of EGFR antibody, Cetuximab (C) with gemcitabine and oxaliplatin (GEMOX) was tested in advanced BTC patients. In the BINGO trial, C-GEMOX therapy achieved a median progression-free survival (PFS) of 6.1 compared to 5.5 months in GEMOX arm [13, 14]. Further trials investigated the potential role of K-RAS mutational status as a predictor of response to EGFR therapies (Cetuximab or Panitumumab) in BTC. Results demonstrated only a marginal benefit in K-RAS wild type (WT) patients versus mutated in terms of survival [15, 16]. The Vecti-BIL study, which selected K-RAS WT patients in a randomized study of GEMOX plus Panitumumab (P) vs GEMOX alone, confirmed the marginal role of Panitumumab (P) in improving PFS (5.3 months in P-GEMOX arm vs 4.4 months in GEMOX arm) and no benefit in overall survival (OS) [17].

Recent data on colorectal cancer suggested that one of the molecular mechanisms responsible of primary and acquired resistance to anti-EGFR therapies is the activation of MEK/MAPK [18–20] and that the simultaneous inhibition of EGFR and MEK/MAPK could overcome this resistance. The inhibition of EGFR downstream transducer was already tested in a phase II trial with the MEK inhibitor Selumetinib as single agent in BTC patients, with an overall response rate (ORR) of 12% [21]. Other MEK inhibitors, such as Trametinib and MEK162, are under investigation in phase I/II ongoing clinical trials in advanced BTC in combination with chemotherapy (NCT02042443; NCT01828034, respectively). To date, no data are available on the simultaneous inhibition of EGFR and MEK/MAPK in BTC.

The strategies of multi-target inhibition focused on vertical signaling pathway targeting (combination treatment with EGFR and MEK1/2 inhibitors) could also synergistically inhibit BTC cancer cell growth.

Here, we investigated the preclinical activity of Panitumumab and Trametinib in BTC preclinical models harboring different K-RAS mutational status.

## RESULTS

### Trametinib inhibits *in vitro* growth of K-RAS mutated BTC cell lines

The preclinical activity of the anti-EGFR Panitumumab, of the MEK inhibitor Trametinib, and of their combination was *in vitro* assessed using seven BTC cell lines expressing basal level of the targets and with different K-RAS mutational status.

Two cell lines, the ICC cell line MT-CHC01 and the extrahepatic cholangiocarcinoma (ECC) cell line EGI-1 are mutated for K-RAS (G12D); the (ECC) WITT and TFK-1 cells, the gallbladder carcinoma (GBC) TGBC1 cells, the (ICC) HUH28 and the ICC mixed to hepatocarcinoma KMCH cells were K-RAS WT. IC50 values showed that the K-RAS mutated cell lines were sensitive to Trametinib, with an IC50 of 3.12 and 6.25 nM, respectively, while the other cells were unresponsive. All the cell lines were insensitive to Panitumumab (IC50> 5 μM) independently from the K-RAS status (Table 1). The combination did not potentiate the effect of Trametinib alone in any cell lines (data not shown).

**Table 1:** IC50 values of drugs in BTC cell lines with different K-RAS genomic status

CELL LINE	TRAM (IC50 nM)	PAN (IC50 μM)
**EGI-1 (ECC) K-RAS G12D**	6.25 nM	>5 μM
**MT-CHC01 (ICC) K-RAS G12D**	3.12 nM	>5 μM
**WITT (ECC) K-RAS WT**	>50 nM	>5 μM
**TFK-1 (ECC) K-RAS WT**	>50 nM	>5 μM
**HUH28 (ICC) K-RAS WT**	>50 nM	>5 μM
**TGBC1 (GBC) K-RAS WT**	>50 nM	>5 μM
**KMCH (ICC MIXED TO HEPATOCARCINOMA) K-RAS WT**	>50 nM	>5 μM

**IC50:** dose of drug which inhibits 50% of the cell growth compared with control calculated for each cell line after 72 hours of treatment; **ECC:** extrahepatic cholangiocarcinoma; **ICC:** intrahepatic cholangiocarcinoma; **GBC:** gallbladder carcinoma; **WT:** wild type. **TRAM**: Trametinib; **PAN**: Panitumumab.

### Trametinib inhibits the activation of MAPK *in vitro*

The next step consists in verifying if the EGFR and MEK-MAPK transduction pathways were inhibited upon Trametinib, Panitumumab, or their combination treatment. We decided to use, as experimental *in vitro* and *in vivo* models, the three tumorigenic cell lines EGI-1, WITT and MTCHC01.

**Figure 1:**
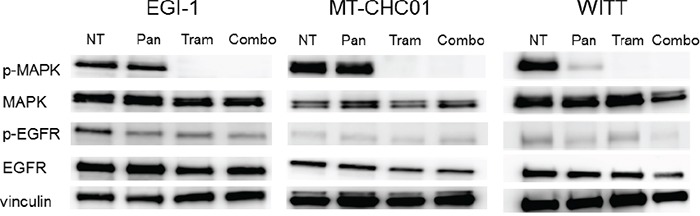
Western Blot analysis for the evaluation of inhibition of Trametinib and Panitumumab targets Cell lines were treated with 50 nM Trametinib (Tram) and 5 μM Panitumumab (Pan) in monotherapy or in combination (Combo) and the expression of p-MAPK, MAPK, p-EGFR, EGFR and Vinculin was investigated.

Cell lines were treated with 50 nM of Trametinib, 5 μM of Panitumumab, or their combination for 3 hours. Western blot analysis (Figure 1) demonstrated that Trametinib was able to switch off the MAPK1,2 activation in all the cell lines, independently by K-RAS status. It is interesting to note that in EGI-1 cells, Trametinib was also able to inhibit EGFR phosphorylation and, even less evident, also in WITT cells. Panitumumab reduced phospho-EGFR expression in EGI-1 cells and slightly in WITT cells. Further, Panitumumab was able to switch the MAPK activation in WITT cells.

### Trametinib slows tumor growth and inhibits angiogenesis in xenograft models of K-RAS mutated BTC

**Figure 2:**
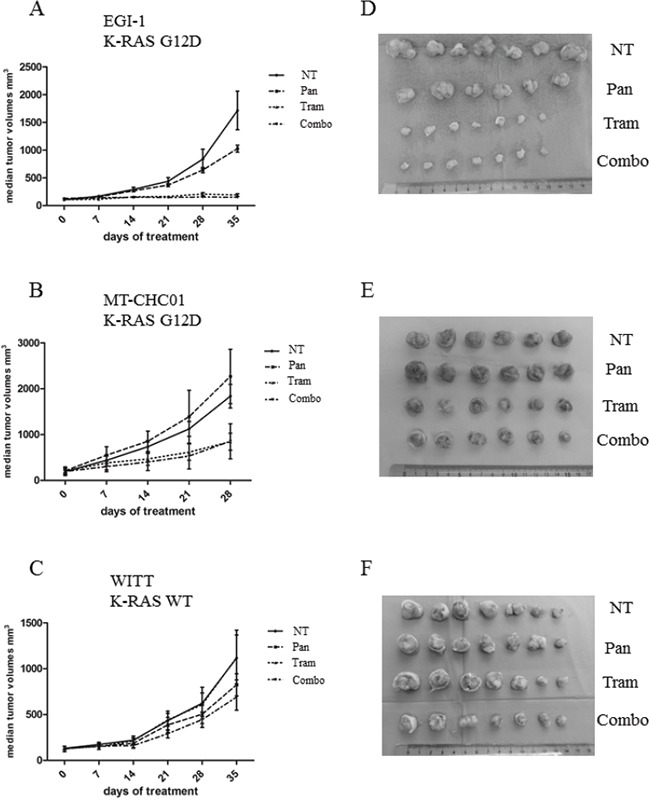
*In vivo* anti-tumor activity of Trametinib and Panitumumab and their combination in human BTC preclinical models The graphs indicate the median tumor volume (mm^3^) weekly measured (**Panel A: EGI-1; Panel B: MT-CHC01; Panel C: WITT):** 0 (start of treatment), 7, 14, 21, 28 and 35 days after treatment with Trametinib (Tram 0.3 mg/kg/die), Panitumumab (Pan 200μg/mouse/twice a week), their combination (Combo), or drug vehicles (NT) (error bars: SD). Seven mice for each arm of treatment in three independent experiments were used. **Panel D, E and F:** representative tumors derived from EGI-1, MT-CHC01 and WITT xenografts, respectively.

Preclinical activity of Trametinib and Panitumumab was also evaluated in EGI-1, MT-CHC01 and WITT xenografts; 5×10^6^ cells were subcutaneously injected in the right flank of 28 mice and four groups (n=7) were created. After two/three weeks, tumors volume reached 100-200 mm^3^. Mice were then randomized to receive different treatments: the first cohort was intraperitoneally treated with Panitumumab (200μg/mouse twice a week), the second cohort orally received (by gavage) Trametinib (0.3 mg/kg/die), another cohort received both drugs, and the last cohort was treated with the drug diluents as a control. Treatment was stopped at the day 28 for MT-CHC01 for their aggressiveness, while for the other two xenografts, treatment was continued up to 35 days. Tumors were calibrated weekly. One day after the last drug administration, mice were sacrificed and tumors were harvested; curves of tumor volumes showed that in xenografts harboring K-RAS mutation, in particular in the EGI-1 xenografts, Trametinib drastically slowed the tumor growth down (p <0.0001) (Figure 2A and 2D) compared to the control arm. In EGI-1, Panitumumab did not significantly potentiate the effectiveness of Trametinib, which appears to be the real player *in vivo*. By the way, Panitumumab in monotherapy slowed the tumor growth in a statistically significant manner compared to the control group (p= 0.0001). In MT-CHC01 xenografts, both Trametinib and combination reduced tumor growth in a statistically significant manner (p<0.0001) at the end of treatment, even if the effect was not marked as in EGI-1 *in vivo* model (Figure 2B and 2E). In K-RAS WT WITT xenografts, only the drug combination slowed the tumor growth (p=0.01) (Figure 2C and 2F).

### Effect of Trametinib and/or Panitumumab on the expression of MAPK, Ki67, and CD31 in BTC in *in vivo* models

**Figure 3:**
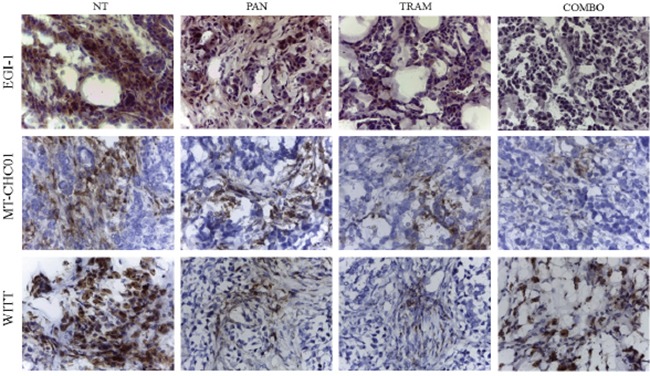
Representative images of immunohistochemistry analysis for the evaluation of MAPK phosphorylation on tumor sections of EGI-1, MT-CHC01 and WITT xenografts treated with Panitumumab (PAN), Trametinib (TRAM), their combination (COMBO) and drug vehicle (NT)

To investigate the mechanism of tumor growth inhibition observed in BTC in *in vivo* models, tumor sections derived from xenografts were assessed for the expression of MAPK phosphorylation by IHC, and for Ki67 e CD31 expression by immunofluorescent analysis. As shown in Figure 3, the phosphorylation of MAPK was reduced in EGI-1 xenografts both by Panitumumab and Trametinib in monotherapy and completely inhibited by the drug combination (as in *in vitro* model). The same result was obtained in MT-CHC01 xenografts, but the drug combination was not able to completely turn the MAPK signaling off. In the WITT xenografts, MAPK activation was predominantly inhibited in Trametinib treated mice, but a slight effect was shown also in Panitumumab-treated cohort. The combination arm reflected the effect of Trametinib alone.

**Figure 4:**
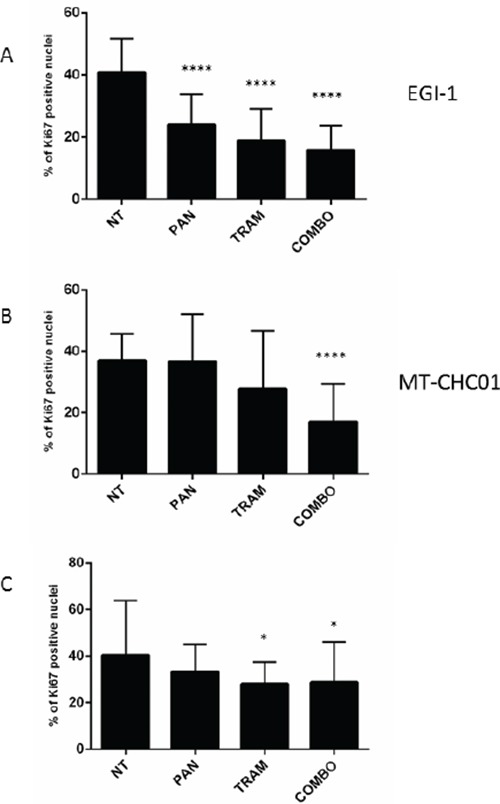
Quantification of Ki67 expression on tumor sections derived from EGI-1 A. MT-CHC01 B. and WITT C. xenografts treated with Panitumumab (PAN), Trametinib (TRAM) and their combination (COMBO). NT: mice treated with drug vehicle

The evaluation of Ki67 expression in EGI-1 xenografts (Figure 4A and Supplementary Figure S1) demonstrated that all treatments induced a significant decrease of tumor proliferation (p <0.0001), although this phenomenon is more evident in the presence of Trametinib and of the drug combination. The quantification of Ki67 positive cells showed a 40.62% proliferation index in not-treated EGI-1 xenograft model; in xenografts treated with Panitumumab and Trametinib in monotherapy there was a decrease of Ki67 positive cells at 23.9% and 18.86% respectively, while the combined treatment further decreased the proliferation rate (15.65%)), consistent with the significant slow of tumor growth. In MT-CHC01 model, Ki67 proliferation index remained unchanged in the presence of Panitumumab (37.00% in untreated vs 36.64% in Panitumumab treated), and decreased to 27.82% and 16.98 with Trametinib and the drug combination, respectively (p< 0.0001 in the drug combination) (Figure 4B and Supplementary Figure S2). Finally, in WITT model, Ki67 index was moderately reduced only in Trametinib and in drug combination compared with not-treated xenografts (40% in NT vs 28% of the treated, p=0.01 (Figure 4C and Supplementary Figure S3).

**Figure 5:**
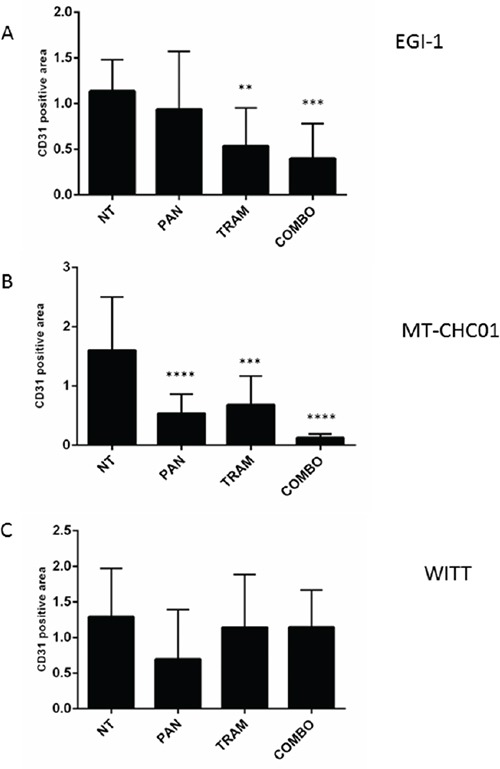
Quantification of CD31 expression on tumor sections derived from EGI-1 A. MT-CHC01 B. and WITT C. xenografts treated with Panitumumab (PAN), Trametinib (TRAM) and their combination (COMBO). NT: mice treated with drug vehicle

To evaluate the antiangiogenic potential of the drugs, xenografts were analyzed for CD31 expression. The results showed that Trametinib and its combination with Panitumumab significantly decreased the vascular density in EGI-1 xenografts (p<0.01 and 0.001 respectively), whereas Panitumumab as single agent had only a moderate effect on tumor angiogenesis (Figure 5A and Supplementary Figure S4). In MT-CHC01 xenografts, the vessel formation was significantly decreased in all treatments (Figure 5B and Supplementary Figure S5) (p<0.001 for Trametinib; p< 0.0001 for Panitumumab and the drug combination). Finally, in WITT xenografts, none of the treatment arms decreased the CD31 expression (p>0.01) (Figure 5C and Supplementary Figure S6).

## DISCUSSION

The use of anti-EGFR therapies in BTC was thoroughly studied in the last years, but results were not encouraging. Primary or acquired resistance is the main obstacle to overcome; one of the mechanisms of failure of anti-EGFR therapies is the presence of downstream pathway mutations, namely K-RAS [15]. The employment of a K-RAS downstream MEK inhibitor could be the key to overcome the resistance to anti-EGFR therapies, as demonstrated in lung ad colon cancer [23, 24]. In this work, we demonstrated that the MEK inhibitor Trametinib, as single agent, significantly reduced the proliferation and the tumor growth in two K-RAS mutated BTC models and is able to contrast the tumor growth in K-RAS WT model in association with Panitumumab.

K-RAS is one of the most commonly mutated genes in BTCs, but the prevalence of these alterations varies widely among studies and is related to ethnicity. In a retrospective study of case series of 153 surgically-resected primary biliary cancers (including 70 ICC, 57 ECC and 26 GBC) derived from patients of Italian origin, the multigene next generation sequencing (NGS) analysis demonstrated that 43/153 (28.1%) of patients presented K-RAS mutation; in particular, subdividing for histological groups, 47.4% of ECC shared K-RAS mutations, while ICC and GBC 15.7% and 19.2%, respectively [25]. Our group demonstrated in a cohort of 49 BTC patients that 6.1 % of patients harbored K-RAS mutations [6]. These mutations could support a constitutive activation of MAPK-MEK pathway, suggesting of being a suitable target. To date, the MEK1/2 inhibitor Selumetinib was investigated in advanced BTC patients, not selected for K-RAS mutations, showing an overall response rate of 12%, a PFS of 3.7 months and an OS of 9.8 months [21]. We showed that, *in vitro*, Trametinib was able to switch off the MAPK1,2 phosphorylation in all the cell lines, independently from the basal level of MAPK activation and from the K-RAS mutational status. Only in the two K-RAS mutated cell lines, EGI-1 and MT-CHC01 cells, this inhibition resulted in a reduction of cell growth. These data suggest a key role of MAPK/MEK pathway in the BTC proliferation process. This is in line with other *in vitro* results described by Jing and colleagues, who evaluated the efficacy of Trametinib in a panel of 218 tumor cell lines, demonstrating a correlation between the presence of K-RAS mutations and drug response [26]. On the contrary, the K-RAS WT WITT cells are resistant to the MEK inhibitor.

Despite the wild type K-RAS mutational status of WITT, they are resistant not only to Trametinib, but also to Panitumumab *in vitro* at 72 hrs and up to 144 hrs of treatment. In a similar study, in a K-RAS WT BTC cell line, the TFK-1, it has been demonstrated that Cetuximab decreased *in vitro* cell growth in a dose dependent manner only after twelve days of treatment and in sub-cultured cells [27].

The *in vivo* data reflect those obtained *in vitro*, demonstrating that Trametinib alone is able to slow tumor growth inhibiting both proliferation and angiogenesis in K-RAS mutated xenografts. Horiuchi and collaborator previously showed that the MEK/ERK inhibitor U0126 was able to prolong survival in gallbladder carcinoma xenograft model with K-RAS mutation [28]. Unexpectedly, a moderate but significant efficacy of Panitumumab was revealed in EGI-1 K-RAS mutated xenograft, suggesting that other mechanisms may be on the basis of resistance to anti-EGFR therapies in BTC. Ki67 expression was reduced by Trametinib in both models, even statistically significant only in EGI-1. The inhibition is potentiated by Panitumumab and Trametinib combination. Concerning the anti-angiogenetic inhibition, CD31 endothelial markers was reduced in all arms of treatment in K-RAS mutated xenografts; for EGI-1 model, the trend of reduction was also evident in the presence of Panitumumab alone, although not significant, due to the high variability. On the contrary, in MTCHC01 xenografts, Panitumumab significantly reduced angiogenesis, but promoted the tumor growth. These data suggest that angiogenesis reduction could be an underlying anti-tumor mechanism in EGI-1 xenograft model; consequently, the two mechanisms are independent and the inhibition of angiogenesis is not a sufficient mechanism to inhibit growth tumor.

The antiangiogenic role of another MEK inhibitor, Selumetinib, was previously reported in lung cancer cells resistant to anti-EGFR therapies [29]. Further, Trametinib demonstrated an anti-angiogenetic activity also in HUVEC cells but not in *in vivo* model of renal cell carcinoma [30].

In K-RAS WT WITT cell line, the drug combination is essential to slow down tumor growth. The angiogenesis inhibition is greater in the presence of Panitumumab alone, which is able to reduce tumor growth, even if not in a statistically significant manner; in contrast, the inhibition of angiogenesis is lower in the presence of the drug combination that exerted the maximum effect in term of tumor growth inhibition. A Ki67 reduction was revealed in the combination arm, in line with tumor growth curves.

In conclusion, our preclinical data suggest that Trametinib could be a promising alternative target therapy for the subgroup of BTC patients harboring K-RAS mutations and for K-RAS WT BTC patients with primary or acquired resistance to anti-EGFR therapies, guaranteeing a strong rational for planning phase II clinical trials.

## MATERIALS AND METHODS

### Cell lines

The extrahepatic cholangiocarcinoma (ECC) cell lines EGI-1 and TFK-1 (DMSZ-German collection of Microrganisms and Cell Cultures), the intrahepatic cholangiocarcinoma (ICC) cell line HuH28 and the gallbladder carcinoma (GBC) cell line TGBC1 (Cell Bank, RIKEN Bioresource Center Riken Cell Bank, Japan) were cultured in RPMI 1640 containing 10% fetal bovine serum (FBS) (all from Sigma–Aldrich, St. Louis, MO, USA), 100 U/mL penicillin and 100 μg/mL streptomycin (P/S) (Life Technologies Gathersburg, MD). The ECC WITT cells and the ICC mixed to hepatocarcinoma KMCH cells (provided by Dr. Andersen, Laboratory of Experimental Carcinogenesis, National Institutes of Health, Bethesda, Maryland), were cultured in DMEM (Sigma–Aldrich) plus 10% FBS. The authentication of all the cell lines was performed by using Cell_ID system (Promega, Corporation, Madison, WI, USA) comparing their profile with those published on the DMSZ database. The ICC cell line MT-CHC01, established in our laboratory [22], was cultured in KO-DMEM/F12 with 10% FBS, P/S and Hepes buffer.

### Drugs

Trametinib was purchased by Sequoia Research Products (UK), resuspended in dimethyl-sulfoxide (DMSO, Sigma-Aldrich), and stocked in aliquots of (40 mM) stored at −80°C. Panitumumab (20mg/ml) was purchased from Amgen Europe (Netherlands) and stored at 4°C. For the *in vivo* experiments, both drugs were diluted in water.

### Cell growth assay

Cells (3,000/well) were seeded onto 96-well tissue culture plates; after 24 hours, they were treated with escalating doses of Panitumumab (5-0.019 μM) and Trametinib (50-0.19 nM) as single agent or in combination in appropriate complete culture medium for other 72 hours. Cell growth was evaluated with the Cell Titer-Glo® cell viability assay (Promega). All tests were performed in quadruplicate and repeated in three independent experiments. IC50 values, (dose of drug able to inhibit 50% of the cell growth compared with control) were calculated using the CalcuSyn software, based on the Chou-Talalay method.

### Western blot

Cells were lysed in Cell lysis buffer (Cell Signaling Technology, Beverly, USA) and centrifuged at 20,000x*g* for 30 minutes; 20 μg of protein were separated with Mini-Protean TGX Precast Gels, 4-20%, then transferred using Trans-Blot Turbo on nitrocellulose Midi membranes (Biorad). Blots were stained using standard procedures and signals were revealed by a chemiluminescence reagent (Euroclone, Milan, Italy). Horseradish peroxidase (HRP)-linked secondary antibodies, anti-phospho-MAPK, anti-MAPK, anti-phospho EGFR, anti-EGFR, anti-vinculin, are from Cell Signaling (Euroclone).

### Antitumor activity of panitumumab and trametinib in *in vivo* models of BTC

NOD female mice (Non-Obese Diabetic) /SCID (Severe Combined immunodeficient) 4-6 weeks (Charles River Laboratory) were maintained under sterile conditions in micro-isolator cages at the animal facilities of the IRCCS-Candiolo. All animal procedures were approved by the Institutional Ethical Committee for Animal Experimentation (Fondazione Piemontese per la Ricerca sul Cancro) and by the Italian Ministry of Health. In three independent experiments, mice were subcutaneously injected with EGI-1, WITT, and MT-CHC01 cells (5×10^6^ cells/mouse) resuspended in 50% (V/V) growth factor–reduced BD Matrigel basement membrane matrix (BD Bioscience). When tumors reached a volume of 100-200 mm^3^ (about 2 weeks after injection), animals were divided into four arms of treatment: a cohort of seven mice were treated daily with Trametinib (0.3 mg/kg/die) by oral gavage, another cohort received Panitumumab (200 μg per mouse) twice a week intraperitoneally, a cohort received the combination of the drugs, while the last cohort of mice was treated with the vehicle of the drugs as a control. The treatment was prolonged up to 28-35 days.

Subcutaneous xenograft diameters were measured every 7 days. At the end of the treatment, mice were euthanized, tumor diameters measured, and volumes calculated using the following formula: *V*=*A*×*B*2/2 (*V*= tumor volume, *A*= largest diameter, *B*= smallest diameter). Mean volumes of treated and untreated xenografts were compared using thet wo-way Anova statistics, applying the Bonferroni test, considering as statistically significant a *p*- value less than 0.05 (confidence interval 95%). Tumors were explanted, frozen in OCT or formalin-fixed, paraffin-embedded (FFPE) and processed for the immunohistochemistry and immunofluorescence analyses.

### Immunohistochemistry (IHC) and immunofluorescence on BTC in *in vivo* models

For the evaluation of Panitumumab and Trametinib effect as single agents or in combination, tissues derived from xenografts were stained with anti-Ki67/MIB1 (Dako) and anti-CD31 (BecktonDikinson) antibodies, followed by incubation with secondary antibody (Invitrogen). Ki67 expression was evaluated in 10 fields for each section at 40X by ImageJ; CD31 quantification was performed on 10 z-stack images for each slide at 20x magnification by calculating the positively stained vessel area. Expression values of treated and untreated xenografts were compared by one-way Anova test, assuming a p-value <0.05 (C.I. 95%) as statistically significant. For the evaluation of MAPK activation, the primary polyclonal antibody anti p-MAPK Thr 202/204 was used. The EnVision kit (Dako) was used for the detection.

## SUPPLEMENTARY FIGURES


