# Supplementary material for: Preclinical activity of EGFR and MEK1/2 inhibitors in the treatment of biliary tract carcinoma
Source: Oncotarget. 2016 Jul 13;7(32):52354–63. doi: 10.18632/oncotarget.10587 (PMC5239557; doi:10.18632/oncotarget.10587)
Supplement: Supplementary file 1 [file oncotarget-07-52354-s001.pdf]

Preclinical activity of EGFR and MEK1/2 inhibitors in the treatment of biliary tract carcinoma

SUPPLEMENTARY FIGURES

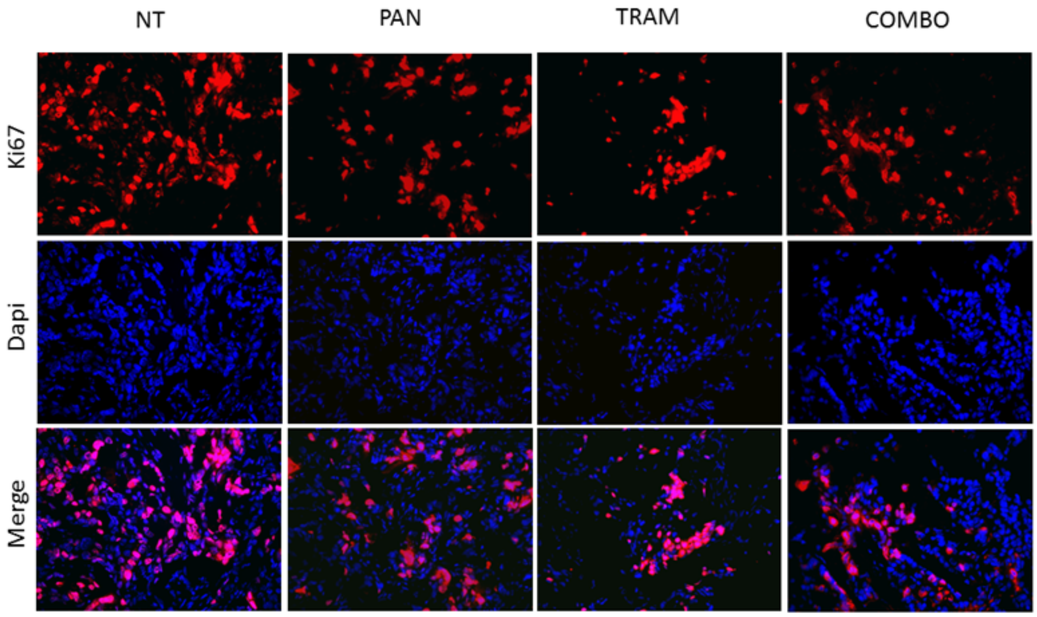

Supplementary Figure S1: Representative images of immunofluorescence analysis for the evaluation of Ki67 proliferation marker on tumor sections derived from EGI-1 xenografts treated with Panitumumab (PAN), Trametinib (TRAM) and their combination (COMBO). NT: mice treated with drug vehicle.

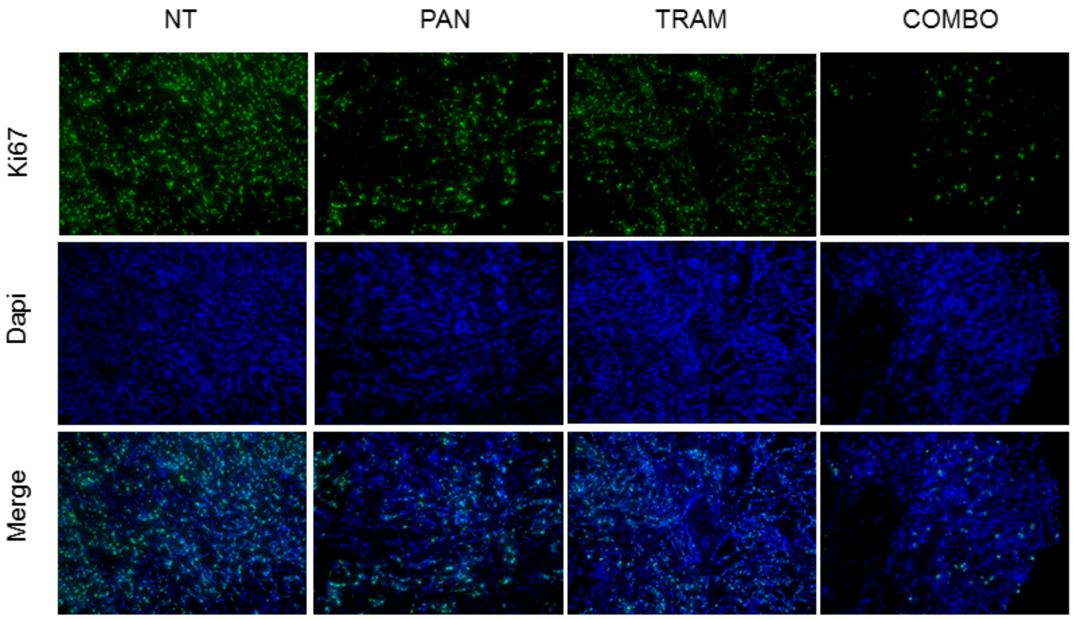

Supplementary Figure S2: Representative images of immunofluorescence analysis for the evaluation of Ki67 proliferation marker on tumor sections derived from MT-CHC01 xenografts treated with Panitumumab (PAN), Trametinib (TRAM) and their combination (COMBO). NT: mice treated with drug vehicle.

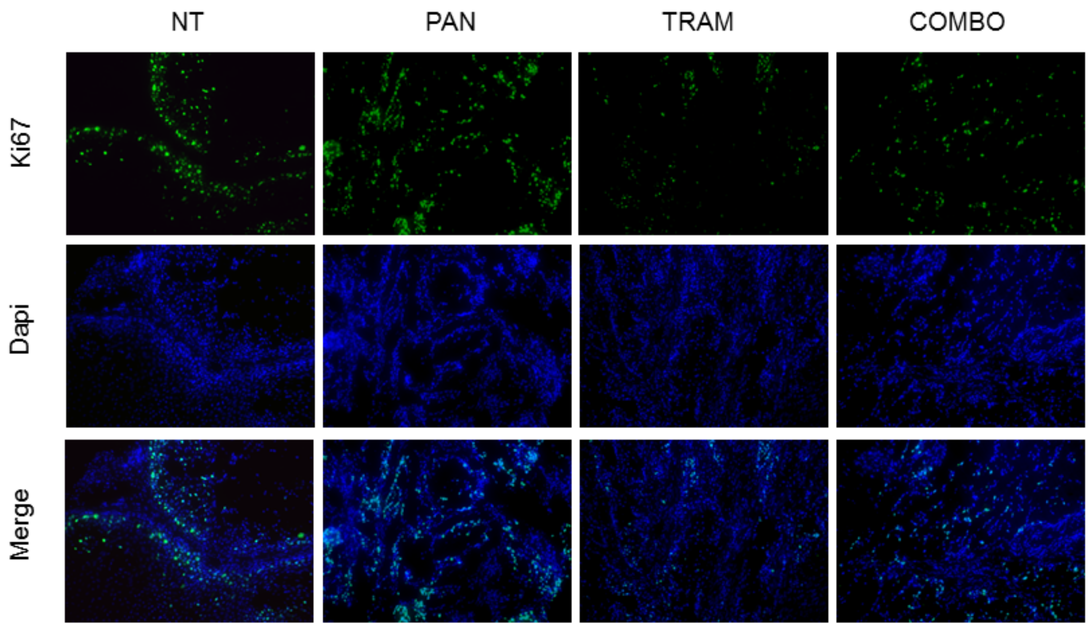

Supplementary Figure S3: Representative images of immunofluorescence analysis for the evaluation of Ki67 proliferation marker on tumor sections derived from WITT xenografts treated with Panitumumab (PAN), Trametinib (TRAM) and their combination (COMBO). NT: mice treated with drug vehicle.

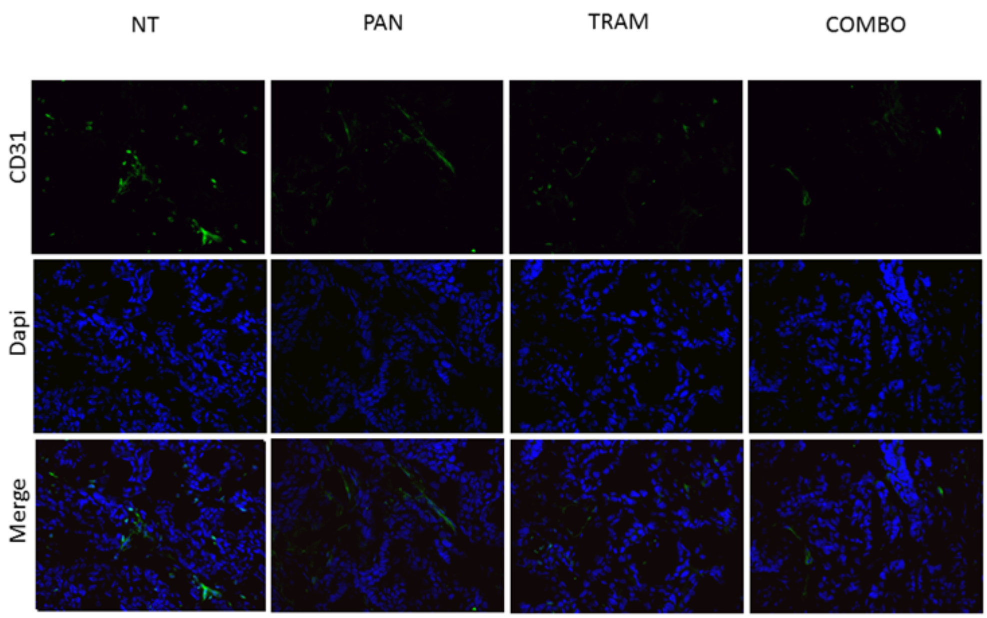

Supplementary Figure S4: Representative images of immunofluorescence analysis for the evaluation of CD31 expression on tumor sections of EGI-1 xenografts treated with Panitumumab (PAN), Trametinib (TRAM) and their combination (COMBO). NT: mice treated with drug vehicle.

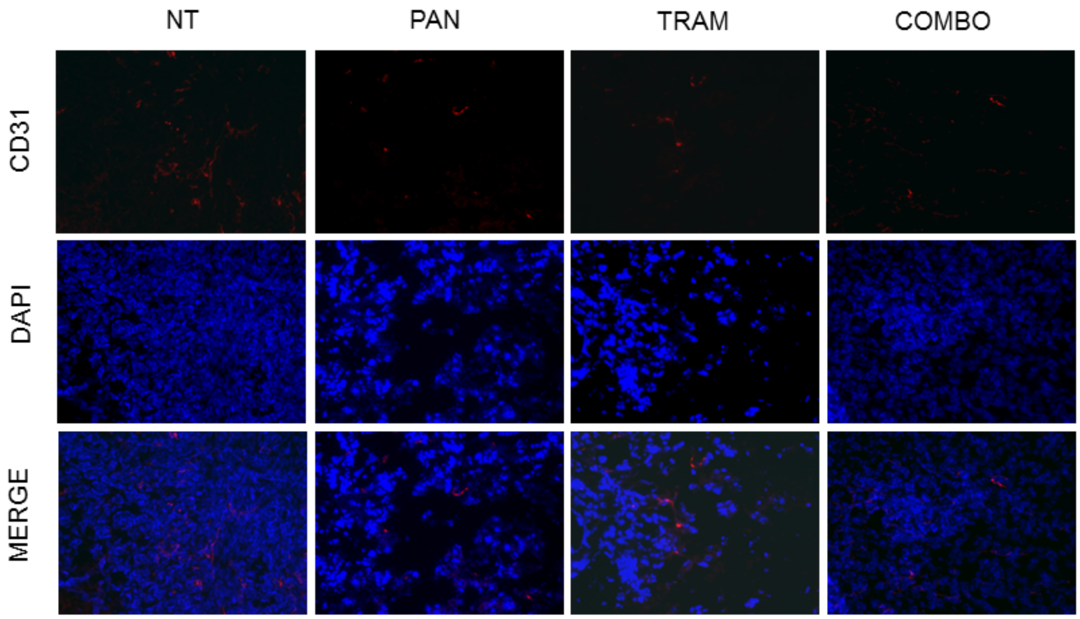

Supplementary Figure S5: Representative images of immunofluorescence analysis for the evaluation of CD31 expression on tumor sections of MT-CHC01 xenografts treated with Panitumumab (PAN), Trametinib (TRAM) and their combination (COMBO). NT: mice treated with drug vehicle.

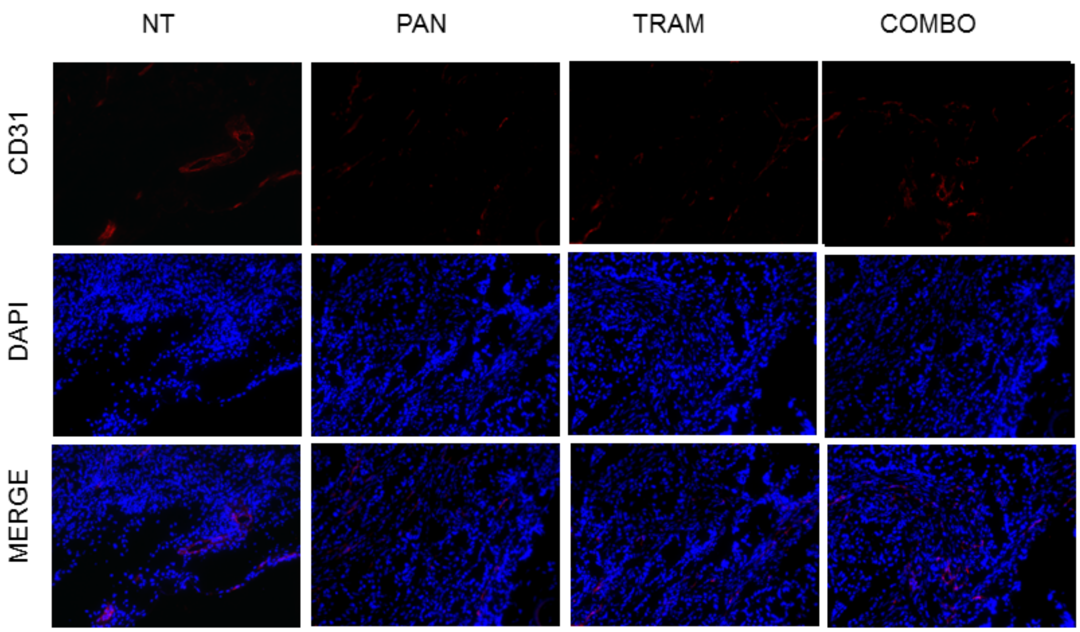

Supplementary Figure S6: Representative images of immunofluorescence analysis for the evaluation of CD31 expression on tumor sections of WITT xenografts treated with Panitumumab (PAN), Trametinib (TRAM) and their combination (COMBO). NT: mice treated with drug vehicle.
